# Real-world quality-of-life of patients with HR+/HER2− advanced breast cancer treated with palbociclib plus endocrine therapy: EORTC QLQ-C30 results from POLARIS

**DOI:** 10.1007/s10549-024-07524-2

**Published:** 2024-11-25

**Authors:** Gabrielle B. Rocque, Joanne L. Blum, Yan Ji, Timothy Pluard, John Migas, Shailendra Lakhanpal, Erin Jepsen, Eric Gauthier, Yao Wang, Monica Z. Montelongo, Joseph C. Cappelleri, Meghan S. Karuturi, Debu Tripathy

**Affiliations:** 1 University of Alabama at Birmingham, Birmingham, AL USA; 2 Baylor-Sammons Cancer Center, Texas Oncology, US Oncology, Dallas, TX USA; 3 Health Partners Institute, St. Paul, MN USA; 4 Saint Luke’s Cancer Institute, Kansas City, MO USA; 5 Mid-Illinois Hematology &, Oncology Associates Ltd, Normal, IL USA; 6 Saint Vincent’s Birmingham, Birmingham, AL USA; 7 Novant Health, Winston-Salem, NC USA; 8 Pfizer Inc, San Francisco, CA USA; 9 Pfizer Inc, San Diego, CA USA; 10 ICON Plc, Blue Bell, Harrisburg, PA USA; 11 Pfizer Inc, Groton, CT USA; 12 The University of Texas MD Anderson Cancer Center, Houston, TX USA

**Keywords:** HR+/HER2− advanced breast cancer, POLARIS, Palbociclib, Patient-reported outcomes, Quality of life, Real-world evidence

## Abstract

**Purpose:**

To evaluate patient-reported health-related quality-of-life (QoL) in patients with hormone receptor–positive/human epidermal growth factor receptor 2–negative (HR+/HER2−) advanced/metastatic breast cancer (ABC) treated with palbociclib in the longitudinal real-world study, POLARIS.

**Methods:**

Data were prospectively collected from adult patients with HR+/HER2− ABC treated with palbociclib plus endocrine therapy (ET) in routine clinical practice. QoL was assessed with the European Organisation for Research and Treatment of Cancer Quality-of-Life Questionnaire-Core 30 (EORTC QLQ-C30) and reported at baseline and months 6, 12, and 18. Data were expressed as absolute scores at a given time and change from baseline for global QoL and functional/symptom scales. Global Heath Status (GHS)/QoL scores were also determined across 6 patient subgroup categories (e.g., age, visceral disease status). Additionally, the proportions of patients with scores below (functional scales) or above (symptom scales) EORTC-validated thresholds reflecting clinical importance of a health problem were determined.

**Results:**

Among patients treated with palbociclib plus ET (N = 1250) who completed questionnaires at any of the study timepoints, mean GHS/QoL scores at months 6 (69.3), 12 (70.1), and 18 (69.9) were higher than baseline (64.0). Similar trends were observed for functional and symptom scales. Mean GHS/QoL scores over time were consistent across the evaluated subgroups. Decreases in the proportions of patients with clinically important functional impairment/symptoms were observed for most functional/symptom scales from baseline through month 18.

**Conclusion:**

Findings from this real-world study indicate patients with HR+/HER2− ABC treated with palbociclib plus ET maintain their QoL for at least 18 months.

Clinical Trial Registration: NCT03280303; registered 12 September 2017

**Supplementary Information:**

The online version contains supplementary material available at 10.1007/s10549-024-07524-2.

## Introduction

Based on clinical trial evidence of improvements in progression-free survival (PFS) without a significant decline in health-related quality of life (QoL), cyclin-dependent kinase 4/6 (CDK4/6) inhibitors in combination with endocrine therapy (ET) are considered the standard of care in patients with hormone receptor–positive/human epidermal growth factor receptor 2–negative (HR+/HER2−) advanced or metastatic breast cancer (ABC) [1–6]. Clinical trials provide critical evidence regarding treatment efficacy, safety, and impact on QoL of new therapies in advanced cancers [6–8]. However, owing to strict eligibility criteria, clinical trials do not fully reflect the outcomes and experience of patients in routine care, thereby limiting generalizability of trial findings [7–9].

To date, there remains a paucity of robust, prospective real-world studies on the QoL of patients receiving newer biological therapies, such as CDK4/6 inhibitors, that have the potential to help guide patient and provider expectations on the impact of treatment [6, 10]. Thus, POLARIS [11], a longitudinal study, was designed to prospectively collect data of patients with HR+/HER2− ABC receiving treatment with palbociclib in routine clinical practice. The aim of this study was to evaluate longitudinal patient-reported QoL outcomes in patients who participated in POLARIS and received palbociclib in the real-world setting and to assess for consistency of study findings across patient subgroups often underrepresented in clinical trials.

## Methods

### Study design

POLARIS (NCT03280303) was a prospective, observational, multicenter, real-world study conducted in > 100 sites in the US (majority of sites) and Canada according to each site’s routine clinical practice and reviewed and approved by applicable local institutional review boards (enrollment: 1 January 2017 − 30 September 2019); study design is shown in Online Resource 1). Detailed methods have been previously published [11].

### Patient population

POLARIS enrolled adult patients (≥ 18 years of age) with a diagnosis of HR+/HER2− ABC who received palbociclib (treatment indication determined by a physician) as first-line (1L), second-line (2L), or later line of therapy (LOT). Study inclusion/exclusion criteria are provided in Online Resource 2. Patients were followed from initiation of palbociclib treatment up to 3 years after the end of treatment, patient study withdrawal, or death, whichever came first. Patient characteristics, treatment, and QoL data were collected from routine clinical assessments performed by treating physicians.

### Patient-reported QoL assessments

One of the outcomes of interest for this analysis was to evaluate patient-reported assessment of global health status (GHS)/QoL using the European Organisation for Research and Treatment of Cancer Quality-of-Life Questionnaire Core 30 (EORTC QLQ-C30) version 3 questions, #29: “How would you rate your overall health during the past week?” and #30: “How would you rate your overall quality of life during the past week?” [12, 13]. Additional outcomes evaluated included patient-reported assessment of five functional scales (physical, role, social, emotional, and cognitive) and nine symptom scales (fatigue, pain, nausea/vomiting, insomnia, appetite loss, constipation, dyspnea, diarrhea, and financial difficulties) on the EORTC QLQ-C30. EORTC QLQ-C30 data were collected and analyzed at baseline (i.e., date of study enrollment when informed consent was obtained and inclusion/exclusion criteria assessed) and at months 6, 12, and 18 for those patients still receiving palbociclib treatment.

Proportions of patients with clinically important impairments/symptoms on the functional and symptom scales of the EORTC QLQ-C30, as defined by the EORTC Quality of Life Group [14], were also determined.

### Statistical analyses

Descriptive analyses of EORTC QLQ-C30 data were performed on the overall study population (N = 1250 patients) and were repeated for the per-label population (n = 861), consisting of patients with HR+/HER2− ABC in the study population who were treated as per the US label indication, defined as palbociclib plus aromatase inhibitor in the 1L setting or palbociclib plus fulvestrant after prior ET in any setting [15]. Patient characteristics were summarized descriptively and were reported for both study populations. For the overall study population, discontinuation of palbociclib and study withdrawal, with reasons, are reported.

Completion rates of the GHS/QoL domain of the EORTC QLQ-C30 were determined at baseline and months 6, 12, and 18. Patient characteristics were additionally summarized for those patients who completed at least the GHS/QoL domain at months 6, 12, and 18. Multivariable logistic regression analyses were conducted to determine the impact of key patient characteristics (e.g., age at enrollment, race/ethnicity, time from diagnosis to enrollment) on the probability of completing the GHS/QoL domain of the EORTC QLQ-C30 at study time points.

For GHS/QoL and functional and symptom scales, mean scores and standard deviations (SD) were reported at baseline and months 6, 12, and 18. For all 15 components, patient responses were converted to a 0–100 scale using the standard EORTC scoring algorithm [16]. A higher score for GHS/QoL and functional scales represents a high QoL and high level of functioning, whereas for symptom scales, a higher score represents a high level of symptomatology. Missing (not completed) data on the questionnaires were handled as described in Online Resource 3. For all components of the EORTC QLQ-C30, a sensitivity analysis of mean scores was conducted that only included patients with completed questionnaires at baseline throughout month 18. Additionally, a statistical analysis of the differences in mean scores for GHS/QoL for various combinations of questionnaire completers versus non-completers was conducted using two-group independent sample t-tests. Mean EORTC QLQ-C30 scores were also compared with the US population normative data [17].

Pre-planned subgroup analyses of GHS/QoL scores by age (< 70 versus ≥ 70 years), race/ethnicity (Black, Indigenous, and People of Color [BIPOC] versus White/Not Hispanic), visceral disease at baseline (yes/no), bone-only metastases at baseline (yes/no), LOT (1L versus ≥ 2L), and any dose modification(s) (yes/no) were conducted. Significant differences in GHS/QoL mean scores of the subgroups at each time point were determined based on two-group independent sample t-tests [16].

Changes from baseline in mean scores to months 6, 12, and 18 were determined for the GHS/QoL, functional, and symptom scales; a ≥ 10-point change in mean score was considered clinically meaningful [18]. Statistical comparisons were made based on paired t-tests of mean changes from baseline [16].

Lastly, EORTC QLQ-C30 data were analyzed as the count and percentage of patients with a health problem (functional impairment/cancer-related symptoms) at baseline, and at months 6, 12, and 18 with functional scale scores below the clinical importance threshold and with symptom scale scores above the clinical importance threshold [14].

## Results

### Patient baseline characteristics

Between January 2017 and January 2023, 1250 patients were enrolled and received at least one dose of palbociclib. Median time from ABC diagnosis to study enrollment was 1.3 months, median age was 64.0 years, 98.8% were female, 81.8% were White, and 11.1% were Black (Table 1). Among the study population, 94.9% had metastatic disease at time of enrollment and 5.0% had locally advanced disease. Among the patients with metastatic disease, 41.7% had visceral disease and 34.1% had bone-only disease. Most of enrolled patients (72.1%) received palbociclib in the 1L setting (median duration of treatment was 14.2 months); 15.0% and 13.0% received palbociclib as 2L and > 2L, respectively (median duration of ≥ 2L treatment was 9.2 months).

**Table 1:** Patient baseline demographic and disease characteristics

Characteristics	Patients (N = 1250)
Age at enrollment, years	
**Median (range)**	64.0 (22–97)
Distribution, n (%)	
< 50	193 (15.5)
50–69	640 (51.4)
≥ 70	413 (33.1)
Sex, n (%)	
Male	15 (1.2)
Female	1235 (98.8)
Race, n (%)	
American Indian or Alaska Native	8 (0.6)
Asian	23 (1.8)
Black	139 (11.1)
Native Hawaiian or other Pacific Islander	5 (0.4)
White	1022 (81.8)
Other	22 (1.8)
Not reported/missing	31 (2.5)
Ethnicity, n (%)	
Hispanic or Latino	106 (8.5)
Not Hispanic or Latino	1106 (88.5)
Not reported/missing	38 (3.0)
Disease stage at enrollment, n (%)	
Locally advanced	62 (5.0)
Metastatic	1186 (94.9)
Not reported	2 (0.2)
Site of distant metastases at ABC diagnosis^a^ Visceral disease	
	494 (41.7)
Bone-only	405 (34.1)
Bone plus other metastases	481 (40.6)
Disposition at enrollment, n (%)	
Recurrent from earlier stage, stages 0–III	849 (67.9)
De novo, stage IV at/near initial diagnosis	341 (27.3)
Not reported	60 (4.8)
Time from ABC diagnosis to enrollment, months	
Median (range)	1.3 (0–248)
Missing, n	4
Distribution, n (%)	
≤ 1 month	514 (41.3)
> 1–2 months	245 (19.7)
> 2–6 months	128 (10.3)
> 6 months	359 (28.8)
Line of therapy^b^, n (%)	
1L	901 (72.1)
2L	187 (15.0)
> 2L	162 (13.0)

^a^Among patients with metastatic disease at study enrollment. Visceral disease refers to metastases of the brain, liver, and/or lung/pleura. ^b^Line of therapy (LOT) is defined as the number of systemic therapies taken after initial diagnoses of advanced or metastatic breast cancer, but before palbociclib treatment start. First-line patients had no LOT before palbociclib initiation. *1L* first-line, *2L* second-line, *> 2L*, greater than second-line, *ABC*, advanced or metastatic breast cancer

### Discontinuation and withdrawal

Among the study population, 85.4% (n = 1068) of patients discontinued palbociclib over the study course. Reasons for treatment discontinuation included disease progression (60.4%), toxicities/side effects (9.7%), patient decision (8.8%), patient physical status (3.0%), completion of planned treatment course (2.3%), poor adherence (0.8%), developed resistance to ET (0.3%), and other (13.7%) or unknown reason (0.9%). After palbociclib discontinuation, patients were followed for a median of 2.9 months; at the end of this follow-up period, 95.6% (n = 1195) of patients had discontinued from the study; reasons included death (42.3%), study terminated by sponsor (32.1%), patient no longer willing to participate in study (8.5%), lost to follow-up (3.3%), adverse event (1.7%), and other reason (12.3%).

### EORTC QLQ-C30 completion rates: GHS/QoL domain

Completion rates for the GHS/QoL domain were 93.4% (n = 1167; 83 missing) at baseline, 58.6% (n = 732; 518 missing) at month 6, 38.7% (n = 484; 766 missing) at month 12, and 28.2% (n = 353; 897 missing) at month 18; patient characteristics of each subgroup are shown in Online Resource 4. Generally, the characteristics of patients completing follow-up questionnaires were consistent with those who completed baseline questionnaires (Table 1). From the multivariable regression analyses (Online Resource 5), the most consistent predictive factors of EORTC QLQ-C30 completion included the time from diagnosis date to study enrollment (greater duration versus less) and diagnosis at enrollment (recurrent from earlier stage [stages 0–III] versus de novo stage IV).

### Mean scores on EORTC QLQ-C30 components

**Fig. 1:**
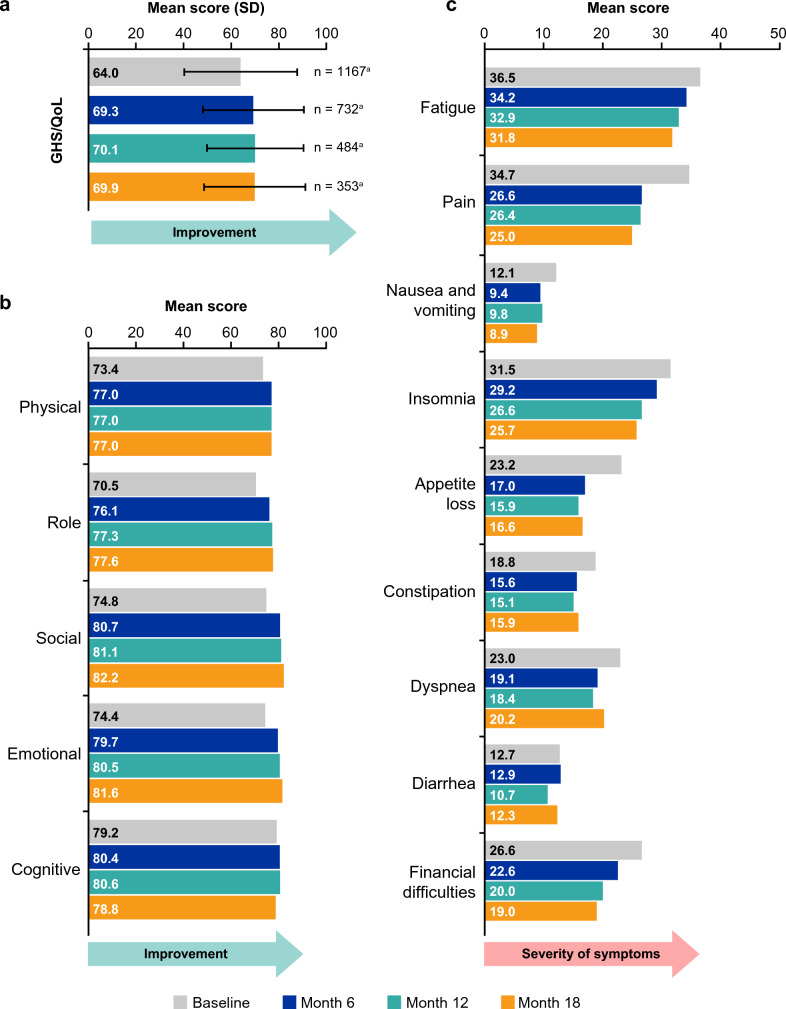
Mean scores of **a** GHS/QoL, **b** functional domains, and **c** symptom domains as measured by the EORTC QLQ-C30. ^a^Mean scores were assessed among the patients with completed EORTC QLQ-C30 questionnaires at the indicated timepoints. Error bars represent standard deviation. Note: a higher score for GHS/QoL and functional scales indicates improvement, whereas for symptom scales, a higher score indicates greater severity of symptoms. EORTC QLQ-C30, European Organisation for Research and Treatment of Cancer Quality-of-Life Questionnaire Core 30; GHS/QoL, global health status/quality of life

Among patients treated with palbociclib plus ET who completed their questionnaires at any of the study time points, mean GHS/QoL scores at months 6 (69.3), 12 (70.1), and 18 (69.9) were higher than recorded at baseline (64.0) (Fig. 1a). The same trend was also observed in the sensitivity analysis conducted with only patients who completed questionnaires at baseline throughout month 18 (n = 246); mean GHS/QoL scores at months 6 (72.9), 12 (71.6), and 18 (69.5) were higher than that recorded at baseline (67.9) (Online Resource 6). Mean score for GHS/QoL among patients who only completed the EORTC QLQ-C30 at baseline was 8.1 points (*P* < 0.001) lower than for patients who completed additional time points beyond baseline (Table 2). Although some significant differences in GHS/QoL scores were found between those who completed and those who did not complete the questionnaire at different time points, these differences were less than the 10-point clinically meaningful threshold (Table 2).

**Table 2:** Differences in mean scores for GHS/QoL among varying levels of questionnaire completion

GHS/QoL completion	Difference in means^a^ (95% CI)^b^	*P*-value^a^
Only at baseline (n = 393) vs at baseline and month 6 (n = 246)	− 4.5 (− 8.4, − 0.6)	0.023
Only at baseline (n = 393) vs at baseline, month 6, and month 12 (n = 162)	− 6.3 (− 10.7, − 1.9)	0.006
Only at baseline (n = 393) vs at baseline, month 6, 12, and 18 (n = 246)	− 8.1 (− 12.0, − 4.3)	< 0.001
At baseline and month 6 (n = 246) vs at baseline, month 6, and month 12 (n = 162)	− 1.8 (− 6.4, 2.8)	0.442
At baseline and month 6 (n = 246) vs at baseline, month 6, 12, and 18 (n = 246)	− 3.7 (− 7.7, 0.4)	0.077
At baseline, month 6, and 12 (n = 162) vs at baseline, month 6, 12, and 18 (n = 246)	− 1.9 (− 6.3. 2.6)	0.411

^a^The difference in means is mean score of fewer measurements (e.g., only at baseline minus mean score of more completed measurements [e.g., at baseline and month 6]). ^b^According to two-group independent sample t-test. CI, confidence interval; GHS/QoL, global health status/quality of life

Among patients treated with palbociclib plus ET who completed their questionnaires at any of the study time points, mean scores of the functional scales also remained stable from baseline to month 18, ranging from 70.5 – 79.2 at baseline, 76.1 − 80.7 at month 6, 77.0 − 81.1 at month 12, and 77.0 − 82.2 at month 18 (Fig. 1b). Likewise, mean scores of the symptom scales remained generally stable with some numerical improvement observed from baseline to month 18 (Fig. 1c). Similar trends were observed in the sensitivity analysis of only patients who completed questionnaires at baseline throughout month 18 (Online Resource 7).

### Comparison with normative data

GHS/QoL scores seen at baseline (64.0) through study month 18 (69.9) were similar or numerically higher than in the general US population (63.9) (Table 3) [17]. These similarities held true for the majority of the functional scales. Regarding mean scores on the symptom scales, in which lower scores indicate less symptom severity, at month 18, pain, nausea/vomiting, insomnia, constipation, and diarrhea were numerically lower than corresponding (US population) normative data.

**Table 3:** Mean EORTC QLQ-C30 scores compared with US normative data

	POLARIS Mean score (SD)	US normative data (n = 1009)^e^ Mean score (SD)
GHS/QoL		
**Baseline**^a^ Month 6^b^ Month 12^c^	64.0 (23.7) 69.3 (21.2) 70.1 (20.2)	63.9 (22.9)
Month 18^d^	69.9 (21.3)	
Functional scales		
Physical Baseline Month 6 Month 12		80.8 (25.2)
	73.4 (24.7) 77.0 (22.1) 77.0 (21.6)	
Month 18	77.0 (22.5)	
Role Baseline Month 6 Month 12	70.5 (32.7) 76.1 (27.8) 77.3 (26.7)	81.7 (28.2)
Month 18	77.6 (27.1)	
Social Baseline Month 6 Month 12 Month 18	74.8 (29.5) 80.7 (25.0) 81.1 (25.2) 82.2 (22.9)	81.6 (29.4)
Emotional Baseline Month 6 Month 12 Month 18	74.4 (23.1) 79.7 (20.3) 80.5 (21.0) 81.6 (18.4)	73.3 (28.0)
Cognitive Baseline Month 6 Month 12 Month 18	79.2 (24.0) 80.4 (21.4) 80.6 (22.9) 78.8 (22.3)	80.9 (25.6)
Symptom scales		
Fatigue Baseline Month 6 Month 12 Month 18	36.5 (26.8) 34.2 (23.1) 32.9 (24.5) 31.8 (24.2)	31.9 (27.8)
Pain Baseline Month 6 Month 12 Month 18	34.7 (31.6) 26.6 (26.3) 26.4 (27.3) 25.0 (27.3)	27.5 (30.2)
Nausea/vomiting Baseline Month 6 Month 12 Month 18	12.1 (20.8) 9.4 (17.3) 9.8 (17.2) 8.9 (16.6)	10.9 (22.6)
Insomnia Baseline Month 6 Month 12 Month 18	31.5 (31.0) 29.2 (28.8) 26.6 (28.8) 25.7 (27.6)	30.8 (33.2)
Appetite loss Baseline Month 6 Month 12 Month 18	23.2 (30.0) 17.0 (25.0) 15.9 (25.4) 16.6 (25.5)	14.1 (25.3)
Constipation Baseline 6 months 12 months 18 months	18.8 (27.4) 15.6 (23.6) 15.1 (23.7) 15.9 (23.6)	18.6 (28.6)
Dyspnea Baseline 6 months 12 months 18 months	23.0 (28.8) 19.1 (24.6) 18.4 (24.4) 20.2 (23.6)	19.9 (28.5)
Diarrhea Baseline 6 months 12 months 18 months	12.7 (23.2) 12.9 (22.6) 10.7 (20.3) 12.3 (22.0)	13.7 (27.1)
Financial difficulties Baseline 6 months 12 months 18 months	26.6 (33.3) 22.6 (30.3) 20.0 (27.8) 19.0 (26.9)	17.5 (30.8)

A higher score for GHS/QoL and functional scales indicates improvement, whereas for symptom scales, a higher score indicates greater severity of symptoms. ^a^n = 1167; ^b^n = 732; ^c^n = 484; ^d^n = 353; ^e^Source of US population norm–Nolte et al., 2019 [17]

EORTC QLQ-C30, European Organisation for Research and Treatment of Cancer Quality-of-Life Questionnaire Core 30; GHS/QoL, global health status/quality of life; SD, standard deviation

### Subgroup analyses

**Fig. 2:**
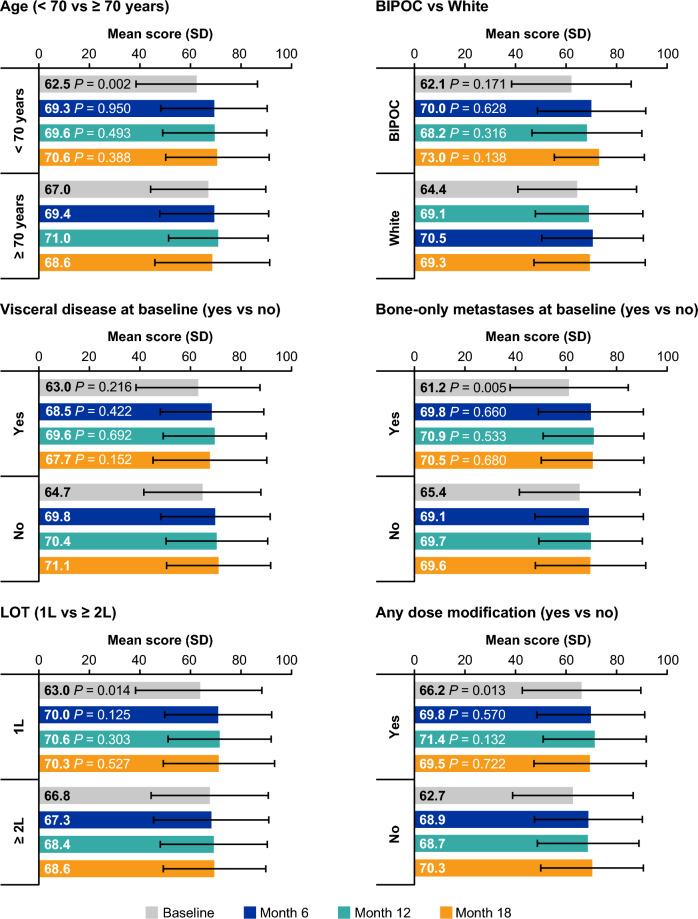
Subgroup analyses of mean scores of GHS/QoL. Error bars represent standard deviation. Significant differences in GHS/QoL mean scores of the subgroups at each time point were determined based on two-group independent sample t-tests, with the corresponding reflecting the difference between subgroups at that particular time point. Mean scores were assessed among patients with completed EORTC QLQ-C30 questionnaires at the indicated timepoints. Age–baseline: < 70 years, n = 767; ≥ 70 years, n = 399; Month 6: < 70 years, n = 485; ≥ 70 years, n = 247; Month 12: < 70 years, n = 321; ≥ 70 years, n = 163; Month 18: < 70 years, n = 230; ≥ 70 years, n = 123. Race – baseline: BIPOC, n = 257; White, n = 867; Month 6: BIPOC, n = 147; White, n = 558; Month 12: BIPOC, n = 94; White, n = 370; Month 18: BIPOC, n = 70; White, n = 270. Visceral disease – baseline: yes, n = 460; no, n = 707; Month 6: yes, n = 268; no, n = 464; Month 12: yes, n = 179; no, n = 305; Month 18: yes, n = 123; no, n = 230. Bone-only metastases – baseline: yes, n = 383; no, n = 784; Month 6: yes, n = 249; no, n = 483; Month 12: yes, n = 169; no, n = 315; Month 18: yes, n = 125; no, n = 228. LOT–baseline: 1L, n = 842; ≥ 2L, n = 325; Month 6: 1L, n = 548; ≥ 2L, n = 184; Month 12: 1L, n = 365; ≥ 2L, n = 119; Month 18: 1L, n = 274; ≥ 2L, n = 79. Any dose modification – baseline: yes, n = 449; no, n = 718; Month 6: yes, n = 330; no, n = 402; Month 12: yes, n = 244; no, n = 240; Month 18: yes, n = 192; no, n = 161. 1L, first-line; ≥ 2L, second-line or later; BIPOC, Black, Indigenous, and People of Color; GHS/QoL, global health status/quality of life; LOT, line of therapy

Across the six subgroup categories analyzed (age at baseline [< 70 versus ≥ 70 years], race [BIPOC versus White], visceral disease at baseline [yes/no], bone-only metastases at baseline [yes/no], LOT [1L versus ≥ 2L], and any dose modification [yes/no]), mean GHS/QoL scores did not significantly differ at months 6, 12, and 18 (Fig. 2).

### Mean changes from baseline on EORTC QLQ-C30 components

**Fig. 3:**
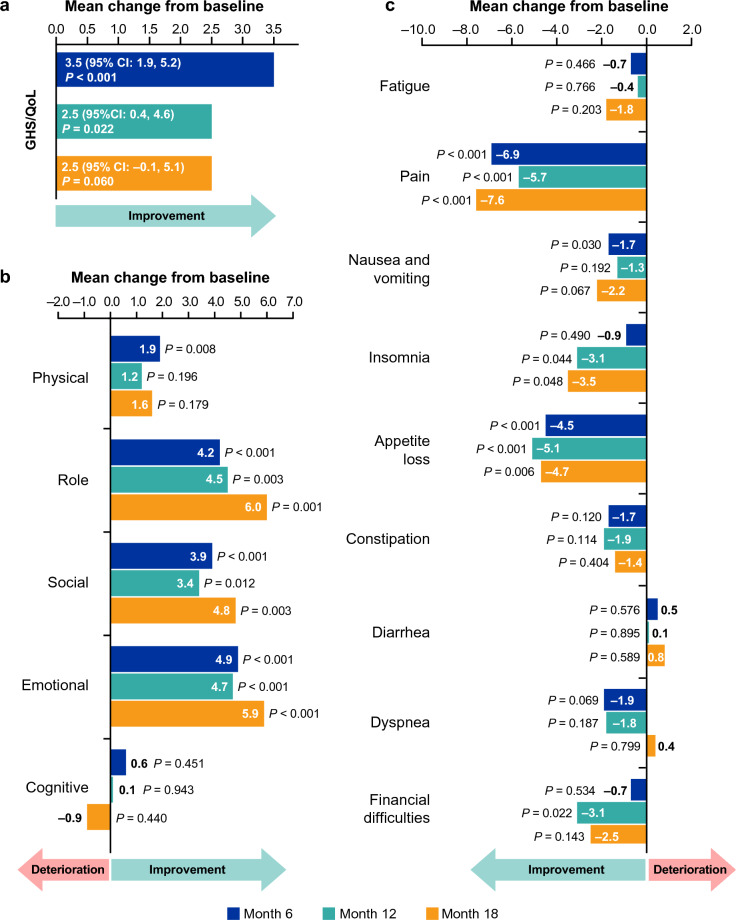
Mean change from baseline in mean scores of **a** GHS/QoL, **b** functional domains, and **c** symptom domains as measured by the EORTC-QLQ-C30 A higher score for GHS/QoL and functional scales indicates improvement, whereas for symptom scales, a higher score indicates greater severity of symptoms (i.e., deterioration). Mean change from baseline represents mean score postbaseline minus mean score at baseline. *P*-values determined from paired t-test of mean changes from baseline. CI, confidence interval; EORTC QLQ-C30, European Organisation for Research and Treatment of Cancer Quality-of-Life Questionnaire Core 30; GHS/QoL, global health status/quality of life

Mean changes from baseline in GHS/QoL scores at months 6 (3.5 [95% CI: 1.9, 5.2], *P* < 0.001) and 12 (2.5 [95% CI: 0.4, 4.6], *P* = 0.022) reached statistical significance, suggesting improvement, but did not reach the 10-point clinically meaningful threshold (Fig. 3a). Mean changes from baseline ranged from − 0.9 to 6.0 across the functional scales and from − 7.6 to 0.8 across the symptom scales (Fig. 3b and c). The largest improvement from baseline was seen in the pain scale, reaching − 7.6 at month 18 (*P* < 0.001), with most of this change already evident by month 6 (− 6.9; *P* < 0.001). None of the mean changes in the scales achieved the 10-point clinically meaningful threshold.

### Proportions of patients with clinically important functional/symptom impairment

The proportions of patients with scores below clinical importance thresholds [14] from baseline up to month 18 numerically trended lower for all functional domains, except cognitive, which was numerically higher by 3.5% (Table 4). For all symptom domains the proportions also trended lower over time, except for dyspnea and diarrhea, which trended slightly upward by 2.5% and 0.5%, respectively (Table 5). The greatest decline was seen in the proportion of patients with clinically important pain, which decreased from 52.9% at baseline to 41.1% at month 18. These trends were relatively consistent when only patients with data reported at every time point were included in the analysis (n = 246; Online Resources 7 and 8).

**Table 4:** Patients with functional scale scores below the clinical importance threshold, suggesting a health problem

Functional scale	Clinical importance threshold [14]	Patients with scores below threshold, n (%)			
		Baseline^a,b^	Month 6^a,b^	Month 12^a,b^	Month 18^a,b^
Physical^c^	< 83	636 (54.4)	368 (50.2)	242 (49.9)	168 (47.3)
Role^c^	< 58	333 (28.5)	147 (20.1)	92 (19.0)	64 (18.0)
Social^d^	< 58	278 (23.8)	110 (15.0)	73 (15.1)	47 (13.3)
Emotional^e^	< 71	430 (36.8)	215 (29.3)	143 (29.6)	97 (27.4)
Cognitive^e^	< 75	395 (33.8)	254 (34.7)	155 (32.0)	132 (37.3)

^a^The number of patients eligible at a visit is based on data expected to be available through the latest date of exposure, visit date, or questionnaire date (baseline, n = 1250; month 6, n = 1100; month 12, n = 978; month 18, n = 834). ^b^Percentages for functional scales were calculated based on “n,” the number of patients with functional score measurements available. ^c^n (missing): baseline, 1170 (80); month 6, 733 (367); month 12, 485 (493); month 18, 355 (479). ^d^n (missing): baseline, 1169 (81); month 6, 732 (368); month 12, 484 (494); month 18, 354 (480). ^e^n (missing): baseline, 1169 (81); month 6, 733 (367); month 12, 484 (494); month 18, 354 (480)

**Table 5:** Patients with symptom scale scores above the clinical importance threshold, suggesting a health problem

Symptom scale	Clinical importance threshold [14]	Patients with scores above threshold, n (%)			
		Baseline^a,b^	Month 6^a,b^	Month 12^a,b^	Month 18^a,b^
Fatigue^c^	> 39	427 (36.5)	258 (35.2)	146 (30.1)	106 (29.4)
Pain^d^	> 25	619 (52.9)	345 (47.1)	224 (46.2)	146 (41.1)
Nausea/vomiting^d^	> 8	424 (36.2)	236 (32.2)	159 (32.8)	114 (32.1)
Insomnia^e^	> 50	280 (24.0)	156 (21.3)	90 (18.6)	62 (17.5)
Appetite loss^e^	> 50	208 (17.8)	72 (9.8)	50 (10.3)	39 (11.0)
Constipation^c^	> 50	141 (12.1)	70 (9.6)	46 (9.5)	28 (7.9)
Dyspnea^f^	> 17	556 (47.6)	327 (44.7)	211 (43.5)	178 (50.1)
Diarrhea^g^	> 17	330 (28.3)	212 (28.9)	124 (25.6)	101 (28.8)
Financial difficulties^h^	> 17	564 (48.4)	326 (44.5)	201 (41.7)	144 (40.8)

^a^The number of patients eligible at a visit is based on data expected to be available through the latest date of exposure, visit date, or questionnaire date (baseline, n = 1250; month 6, n = 1100; month 12, n = 978; month 18, n = 834). ^b^Percentages for symptom scales were calculated based on “n,” the number of patients with symptom score measurements available. ^c^n (missing): baseline, 1170 (80); month 6, 733 (367); month 12, 485 (493); month 18, 354 (480). ^d^n (missing): baseline, 1170 (80); month 6, 733 (367); month 12, 485 (493); month 18, 355 (479). ^e^n (missing): baseline, 1169 (81); month 6, 733 (367); month 12, 485 (493); month 18, 354 (480). ^f^n (missing): baseline, 1167 (83); month 6, 731 (369); month 12, 485 (493); month 18, 355 (479). ^g^n (missing): baseline, 1167 (83); month 6, 733 (367); month 12, 484 (494); month 18, 351 (483). ^h^n (missing): baseline, 1166 (84); month 6, 733 (367); month 12, 482 (496); month 18, 353 (481)

### Patient-reported QoL assessments in the per-label population

The per-label population included 861 patients, representing 68.9% of the overall study population. Among this population, 82.7% received palbociclib in the 1L setting; 8.6% and 8.7% received palbociclib as 2L and > 2L, respectively. Characteristics for this population were generally consistent with the overall study population; notable differences included that a greater proportion were treated with 1L palbociclib (~ 10% more) (Online Resource 9). Online Resource 10 shows the characteristics of patients who completed the EORTC QLQ-C30 at months 6, 12, and 18.

The findings of patient-reported QoL assessments in the per-label population were consistent with that of the overall study population. The difference in mean scores for GHS/QoL among patients who completed the EORTC QLQ-C30 only at baseline versus those who completed it at all time points was − 7.4 (*P* = 0.002; Online Resource 11). Among the patients treated with palbociclib plus ET who completed additional questionnaires at any of the study time points, mean GHS/QoL scores at months 6 (69.7), 12 (71.0), and 18 (69.9) were higher than that recorded at baseline (63.8) (Online Resources 12 and 13). Mean scores of the functional and symptom scales also remained stable from baseline to month 18 (Online Resource 13). Across the six subgroup categories analyzed, mean GHS/QoL scores did not significantly differ at months 6, 12, and 18 (Online Resource 14).

Similar to in the overall study population, mean changes from baseline in GHS/QoL scores at month 6 (5.0 [95% CI: 3.1, 6.9], *P* < 0.001) and 12 (3.5 [95% CI: 1.1, 5.9], *P* = 0.004) reached statistical significance, suggesting improvement, but did not reach the 10-point clinically meaningful threshold (Online Resource 15). Mean changes from baseline in functional and symptom scales also did not exceed the 10-point clinically meaningful threshold (Online Resource 15). Numerically, decreases in the proportions of patients with scores below clinical importance thresholds (suggestive of a health problem) from baseline up to month 18 were observed for all functional domains, except cognitive (Online Resource 16), and for all symptom domains, except dyspnea (Online Resource 17); the proportion of patients with scores below the threshold trended numerically higher in the cognitive domain and in the symptom domain, dyspnea.

## Discussion

The findings of this real-world study indicate that patients with HR+/HER2− ABC treated with palbociclib plus ET in routine clinical practice maintain QoL for at least 18 months of treatment, with GHS/QoL scores averaging 64.0 at baseline and 69.3, 70.1, and 69.9 at months 6, 12, and 18, respectively, while patients continued receiving treatment. Findings among patients treated in routine clinical practice align relatively closely with the results of the PALOMA-3 trial, in which QoL was also measured with the EORTC QLQ-C30 GHS/QoL score [5]. In PALOMA-3, GHS/QoL scores averaged 65.9 at baseline and 66.1 post-treatment among those who received palbociclib plus fulvestrant [5]. Furthermore, our 18-month GHS/QoL data corroborates and extends the reported 36% reduction in risk of QoL deterioration found in PALOMA-3 among patients treated with palbociclib plus fulvestrant relative to those treated with placebo plus fulvestrant after approximately 12 months [5].

The QoL scores observed in POLARIS among patients with HR+/HER2− ABC were also relatively similar to that of US population normative data [17]. A similar observation was made in PALOMA-2 based on Functional Assessment of Cancer Therapy-Breast (FACT-B) normative standards [4]. The real-world patient-reported assessments of QoL while on active treatment with palbociclib in this prospective study provide further evidence of the favorable risk–benefit profile of palbociclib plus ET in the treatment of HR+/HER2− ABC. A recently published systematic review of 15 studies (7 randomized controlled trials, 3 single-arm clinical trials, and 5 real-world studies, including POLARIS study findings reported in published conference abstracts) that evaluated QoL outcomes in patients with HR+/HER2− ABC treated with palbociclib has also concluded that across different study types, populations, palbociclib LOTs, and various QoL measurement instruments, QoL is at least maintained if not improved from baseline in patients treated with palbociclib [19]. These findings with palbociclib are in agreement with the multitude of studies that have evaluated QoL in patients treated with CDK4/6 inhibitors in general, with a systematic review of 31 clinical trials and 7 real-world studies concluding QoL is not adversely impacted by the addition of CDK4/6 inhibitors to ET in patients with estrogen receptor-positive/HER2− ABC [6].

In both of these systematic reviews, it was expressed that it was a challenge to compare QoL results across studies because the main heterogeneity of the studies was the variety of QoL measurement instruments used; however, the EORTC QLQ-C30 was the most prominently used QoL measurement instrument [6, 19]. Currently, there is an international initiative (Setting International Standards in Analyzing Patient-Reported Outcomes and Quality of Life Endpoints Data Consortium) aimed at standardizing design, analysis, presentation, and interpretation of patient-reported outcomes data in cancer research [20, 21]. A recent interview of patients in Europe and the US with different types of cancer, including breast cancer, has found that among participants, the concepts included in the EORTC QLQ-C30 are broadly understood across language versions, and that items included in the instrument are valid and relevant to patients with different cancer sites, stages, and treatments for the assessment of functional health, symptom burden, and QoL [22]. In our study we limited the number of questionnaires to reduce patient burden. Nonetheless, while the generic EORTC QLQ-30 is suitable to assess quality of life in patients with breast cancer, as well as other cancers, we encourage that future research supplement the EORTC QLQ-30 with a disease-specific measure such as the EORTC QLQ-BR45 [23], an improved extension of the QLQ-BR23 that was not available when our study began, or another suitable alternative.

The findings of POLARIS on QoL are encouraging compared with other limited real-world evidence of the QoL in patients with ABC treated mostly with non-targeted therapies. A retrospective QoL analysis of patients with ABC (n = 306) from the German PRAEGNANT registry reported an EORTC QLQ-C30 global QoL score of 56.8 in patients without progression (mean reference for patients with ABC: 60.2 [24]), versus 52.2 in those with progression (n = 65) [25]. At baseline, approximately one-third of patients in this PRAEGNANT cohort had received chemotherapy, known to be associated with lower QoL scores relative to ET [6, 26], and no patients had recorded CDK4/6 inhibitor use [25]. In another real-world QoL study conducted in 5 European countries (70% − 94% treated with ET only), the EORTC QLQ-C30 derived global QoL in women with HR+/HER2− ABC (n = 781) averaged 50.9 [27], significantly lower than the mean reference value of 60.2 for patients with ABC [24], and European general population mean of 72.3 for women aged 60 − 69 years [28]. Interestingly, POLARIS GHS/QoL average scores are also higher than the EORTC QLQ-C30 ABC reference values provided by the EORTC group itself, with published means ranging from 54.6 to 57.6 [29].

In this study, mean changes in EORTC QLQ-C30 functioning and symptom scale scores showed improvement over the course of the study in terms of both mean values (although like GHS/QoL, none crossed the 10-point clinically meaningful threshold) and percentage of patients crossing clinical importance thresholds. The most improvement was seen in the pain scale with an average decrease of 7.6 points by month 18 and 11.8% decline in the proportion of patients above the clinical importance threshold of > 25, which is consistent with findings of the PALOMA-2 and PALOMA-3 QoL analyses [4, 5]. The observed decline in pain may have contributed to the improvements in functioning and overall QoL; further research is warranted. The decline in pain severity that we observed in POLARIS could in part be attributed to 75% of enrolled patients having bone metastases at diagnosis (34.1% bone-only, 40.6% bone plus other metastases). We did find small numerically trending higher proportions of patients over time with health problems in the cognitive domain (3.5%), and symptoms of dyspnea (2.5%) and diarrhea (0.5%). However, mean changes indicating deterioration in these scales were less than 1 point and not considered clinically meaningful.

As with many real-world studies, the interpretation of the findings of this study is subject to limitations. For example, per the observational study design, patient selection and treatment and monitoring procedures were determined by the treating physician in routine clinical practice rather than dictated by a protocol [11], which may have contributed to the fact that approximately one-third of the study population was not treated per-label. The heterogeneous, less-selective patient populations in this study increase the difficulty of interpreting outcomes data [11], and causality cannot be confirmed between treatments and outcomes. Also, it is beyond the scope of these analyses to examine the effects of discontinuation, disease progression, or other factors. Furthermore, the lack of blinding to treatment and missing or inaccurate data on the assessments at follow-up visits must be acknowledged.

Consistent with other prospective real-world studies in the advanced cancers setting [30, 31], there was significant sample attrition in EORTC QLQ-C30 completion over time, which may have led to selection bias due to attrition of the most unwell participants [31]. Of note, 393 of 1250 patients completed the GHS/QoL only at baseline. However, patient characteristics were similar between baseline and all study timepoints analyzed, suggesting that there was no tangible or obvious evidence of subpopulation selection bias. Missing questionnaires (i.e., attrition) is common in real-world studies, usually through negative events experienced by patients (e.g., disease progression, treatment toxicities, death, loss to follow-up, COVID-19), which clearly occurred in POLARIS (i.e., 95.6% of patients withdrew from the study [planned analysis of up to 3 years post-treatment], 42.3% because of death).

Also notable is a survey that reported around a third of POLARIS study sites experienced some impact on their responsiveness to correspondence, timely data entry, or patient management due to the COVID-19 pandemic [32]. We did find in the sensitivity analyses of patients who provided continual completed questionnaire submissions, EORTC QLQ-C30 (GHS/QoL domain) mean values over time were relatively consistent with the main analysis, further suggesting that attrition may have not introduced sizable or dominant bias. Lastly, POLARIS, regarding QoL outcomes, was designed to characterize the QoL of patients who were actively receiving palbociclib treatment, and as patients discontinued palbociclib over the course of the POLARIS study, they no longer completed QoL assessments.

## Conclusion

In this real-world prospective study, among patients with HR+/HER2− ABC and recorded measurements on the EORTC QLQ-C30, QoL was generally maintained while on treatment with palbociclib plus ET. While there was some numerical improvement observed in functioning and symptoms including, most notably, less pain severity, the magnitude of improvement did not reach clinically meaningful thresholds. Overall QoL was also preserved in all evaluated patient subgroups and was found to be fairly equivalent to that in the general US population. These findings are consistent with the patient-reported outcomes and well-tolerated safety profile reported in the PALOMA clinical trials investigating palbociclib plus ET [4, 5, 33, 34].

## Supplementary Information

Below is the link to the electronic supplementary material.

Supplementary file1 (DOCX 774 KB)

## Data Availability

Upon request, and subject to review, Pfizer will provide the data that support the findings of this study. Subject to certain criteria, conditions and exceptions, Pfizer may also provide access to the related individual de-identified participant data. See https://www.pfizer.com/science/clinical-trials/trial-data-and-results for more information.
