# Supplementary material for: Real-world quality-of-life of patients with HR+/HER2− advanced breast cancer treated with palbociclib plus endocrine therapy: EORTC QLQ-C30 results from POLARIS
Source: Breast Cancer Res Treat. 2024 Nov 25;209(3):613–27. doi: 10.1007/s10549-024-07524-2 (PMC11785676; doi:10.1007/s10549-024-07524-2)
Supplement: Supplementary file 1 — Supplementary file1 (DOCX 774 KB) [file 10549_2024_7524_MOESM1_ESM.docx]

## **Supplementary information ─ Online resources**

**Real-world quality-of-life of patients with HR+/HER2− advanced breast cancer treated with palbociclib plus endocrine therapy: EORTC QLQ-C30 results from POLARIS**

Gabrielle Rocque,^1^ Joanne L. Blum,^2^ Yan Ji,^3^ Timothy Pluard,^4^ John Migas,^5^

Shailendra Lakhanpal,^6^ Erin Jepsen,^7^ Eric Gauthier,^8^ Yao Wang,^9^ Monica Z. Montelongo,^10^ Joseph C. Cappelleri,^11^ Meghan S. Karuturi,^12^ Debu Tripathy^12^

^1^University of Alabama at Birmingham, Birmingham, AL, USA; ^2^Baylor-Sammons Cancer Center, Texas Oncology, US Oncology, Dallas, TX, USA; ^3^Health Partners Institute, St. Paul, MN, USA; ^4^Saint Luke’s Cancer Institute, Kansas City, MO, USA; ^5^Mid-Illinois Hematology & Oncology Associates Ltd., Normal, IL, USA; ^6^Saint Vincent’s Birmingham^,^ Birmingham, AL, USA; ^7^Novant Health, Winston-Salem, NC, USA; ^8^Pfizer Inc, San Francisco, CA, USA; ^9^Pfizer Inc, La Jolla, CA, USA; ^10^ICON plc, Blue Bell, PA, USA; ^11^Pfizer Inc, Groton, CT, USA; ^12^The University of Texas MD Anderson Cancer Center, Houston, TX, USA.

**Corresponding author:**

Gabrielle Rocque

University of Alabama at Birmingham

Birmingham, AL, USA

grocque@uabmc.edu

Online Resource 1. POLARIS study design


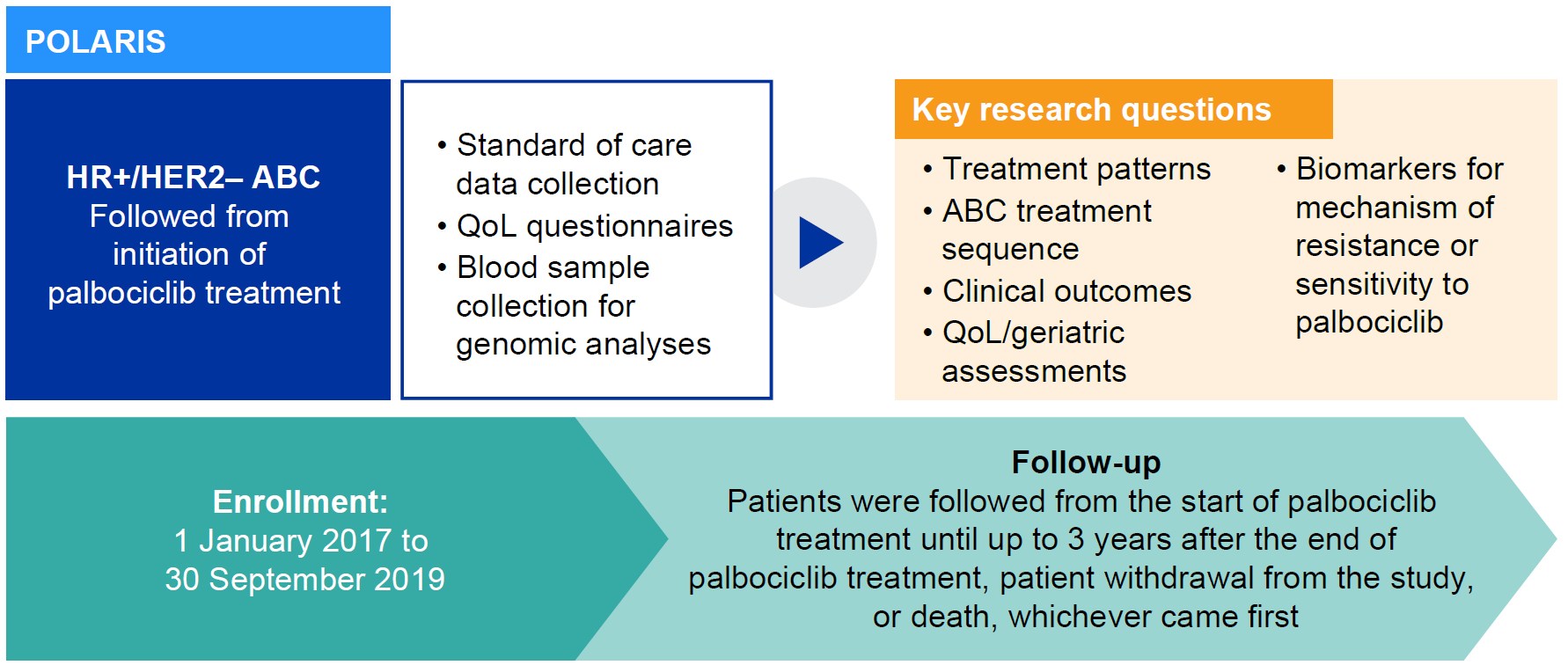


ABC, advanced breast cancer; HR+/HER2−, hormone receptor–positive/human epidermal growth factor receptor 2-negative; QoL, quality of life.

Online Resource 2. POLARIS inclusion and exclusion criteria


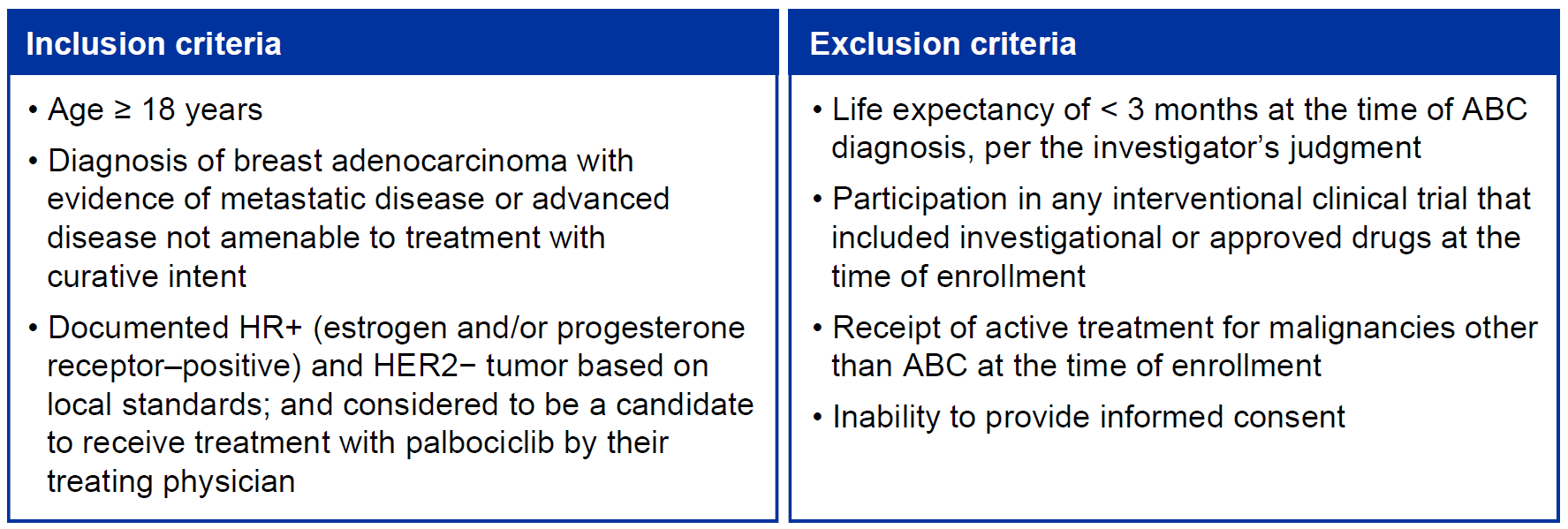
ABC, advanced breast cancer; HER2−, human epidermal growth factor receptor 2-negative; HR+, hormone receptor–positive.

Online Resource 3. Handling of missing data on the EORTC QLQ-C30


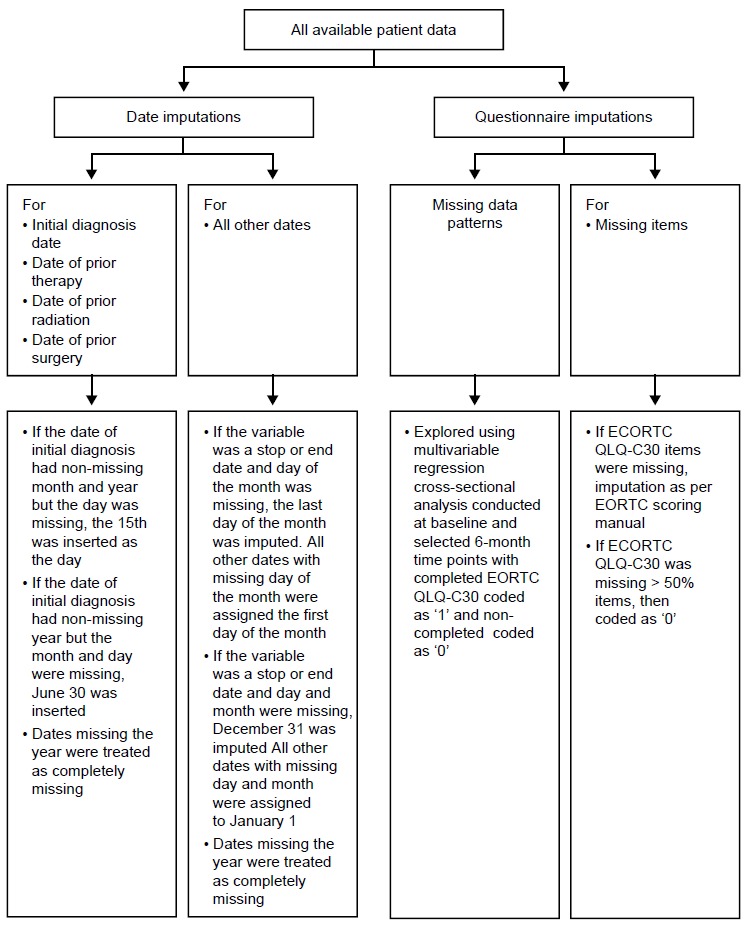


EORTC-QLQ-C30, European Organisation for Research and Treatment of Cancer Quality-of-Life Questionnaire Core 30

**Online Resource 4. Demographic and disease characteristics of patients who completed the EORTC QLQ-C30 (GHS/QoL domain) at study months 6, 12, and 18**

|  | **Month 6** | **Month 12** | **Month 18** |
| --- | --- | --- | --- |
| **Characteristics** | **(n = 732)** | **(n = 484)** | **(n = 353)** |
| Age at enrollment, years | |  |  |
| Median (range) | 65.0 (24.0–96.0) | 65.0 (27.0–88.0) | 65.0 (29.0– 88.0) |
| Distribution, n (%) | |  |  |
| < 50 | 106 (14.5) | 62 (12.8) | 46 (13.0) |
| 50–69 | 379 (51.8) | 259 (53.5) | 184 (52.1) |
| ≥ 70 | 247 (33.7) | 163 (33.7) | 123 (34.8) |
| Sex, n (%) | |  |  |
| Male | 10 (1.4) | 6 (1.2) | 7 (2.0) |
| Female | 722 (98.6) | 478 (98.8) | 346 (98.0) |
| Race, n (%) | |  |  |
| American Indian or Alaska Native | 3 (0.4) | 2 (0.4) | 2 (0.6) |
| Asian | 13 (1.8) | 7 (1.4) | 8 (2.3) |
| Black | 77 (10.5) | 49 (10.1) | 33 (9.3) |
| Native Hawaiian or other Pacific Islander | 3 (0.4) | 3 (0.6) | 1 (0.3) |
| White | 605 (82.7) | 402 (83.1) | 294 (83.3) |
| Other | 12 (1.6) | 7 (1.4) | 6 (1.7) |
| Not reported/missing | 19 (2.6) | 14 (2.9) | 9 (2.5) |
| Ethnicity, n (%) | |  |  |
| Hispanic or Latino | 50 (6.8) | 32 (6.6) | 25 (7.1) |
| Not Hispanic or Latino | 661 (90.3) | 437 (90.3) | 317 (89.8) |
| Not reported/missing | 21 (2.9) | 15 (3.1) | 11 (3.1) |
| Disease stage at enrollment, n (%) | |  |  |
| Locally advanced | 35 (4.8) | 22 (4.5) | 15 (4.2) |
| Metastatic | 696 (95.1) | 461 (95.2) | 338 (95.8) |
| Not reported | 1 (0.1) | 1 (0.2) | 0 |
| Sites of distant metastases at ABC diagnosis^a^ | | | |
| Visceral disease | 268 (38.5) | 179 (38.8) | 123 (36.4) |
| Bone-only | 249 (35.8) | 169 (36.7) | 125 (37.0) |
| Bone plus other metastases | 277 (39.8) | 168 (36.4) | 122 (36.1) |
| Disposition at enrollment, n (%) | |  |  |
| Recurrent from earlier stage, stages 0–III | 480 (65.6) | 306 (63.2) | 220 (62.3) |
| *De novo*, Stage IV at/near initial diagnosis | 212 (29.0) | 148 (30.6) | 111 (31.4) |
| Not reported | 40 (5.5) | 30 (6.2) | 22 (6.2) |
| Time from ABC diagnosis to enrollment, months | |  |  |
| Median (range) | 1.2 (0–248) | 1.3 (0–248) | 1.1 (0–248) |
| Distribution, n (%) | |  |  |
| ≤ 1 month | 313 (42.9) | 200 (41.4) | 162 (46.0) |
| > 1–2 months | 153 (21.0) | 107 (22.2) | 83 (23.6) |
| > 2–6 months | 86 (11.8) | 61 (12.6) | 36 (10.2) |
| > 6 months | 178 (24.4) | 115 (23.8) | 71 (20.2) |
| Line of therapy^b^, n (%) | |  |  |
| 1L | 548 (74.9) | 365 (75.4) | 274 (77.6) |
| 2L | 102 (13.9) | 74 (15.3) | 55 (15.6) |
| > 2L | 82 (11.2) | 45 (9.3) | 24 (6.8) |

^a^ Among patients with metastatic disease at enrollment. Visceral disease refers to metastases of the brain, liver, and/or lung/pleura.

^b^ Line of therapy (LOT) is defined as the number of systemic therapies taken after initial diagnoses of advanced or metastatic breast cancer, but before palbociclib treatment start. First-line patients had no LOT before palbociclib initiation.

1L, first-line; 2L, second-line; > 2L, greater than second-line; ABC, advanced or metastatic breast cancer.

Online Resource 5. Multivariable logistic regression analyses for EORTC QLQ-C30 completion: probability of a patient completing the questionnaire at month 6, month 12 and month 18

| **Characteristic** | **Month 6** | | | | **Month 12** | | | | **Month 18** | | | | |
| --- | --- | --- | --- | --- | --- | --- | --- | --- | --- | --- | --- | --- | --- |
|  | **OR** | **LCL** | **UCL** | ***P*-value** | **OR** | **LCL** | **UCL** | ***P*-value** | | **OR** | **LCL** | **UCL** | ***P*-value** |
| Age at enrollment | 1.004 | 0.993 | 1.015 | 0.514 | 1.011 | 0.999 | 1.022 | 0.063 | | 1.012 | 1.000 | 1.024 | 0.053 |
| White/Not Hispanic (Ref: Black, Indigenous, and People of Color) | 1.255 | 0.911 | 1.729 | 0.165 | 1.333 | 0.955 | 1.862 | 0.092 | | 1.205 | 0.840 | 1.729 | 0.312 |
| Time from diagnosis date to study enrollment distribution (months) | 0.906 | 0.835 | 0.983 | **0.017** | 0.946 | 0.871 | 1.027 | 0.187 | | 0.867 | 0.789 | 0.953 | **0.003** |
| Diagnosis at enrollment (Ref: newly diagnosed stage IV) | 0.841 | 0.622 | 1.138 | 0.262 | 0.730 | 0.539 | 0.987 | **0.041** | | 0.697 | 0.504 | 0.964 | **0.029** |
| Biopsy performed at enrollment (Ref: no) | 1.115 | 0.814 | 1.527 | 0.498 | 1.220 | 0.881 | 1.689 | 0.231 | | 1.191 | 0.832 | 1.703 | 0.340 |
| Visceral disease at enrollment (Ref: no) | 0.660 | 0.473 | 0.920 | **0.014** | 0.835 | 0.599 | 1.165 | 0.289 | | 0.777 | 0.541 | 1.116 | 0.172 |
| Metastases site (Ref: non-bone) | 1.085 | 0.778 | 1.514 | 0.630 | 1.020 | 0.728 | 1.428 | 0.910 | | 0.933 | 0.649 | 1.340 | 0.706 |
| Baseline ECOG PS (Ref: 0) | 0.949 | 0.723 | 1.245 | 0.704 | 0.758 | 0.576 | 0.997 | **0.048** | | 0.945 | 0.702 | 1.272 | 0.710 |
| Number of metastases (Ref: 1) | 1.287 | 0.931 | 1.777 | 0.126 | 0.984 | 0.714 | 1.355 | 0.920 | | 0.929 | 0.659 | 1.309 | 0.672 |
| Hormone partner (Ref: other/none)  Anastrazole  Exemestane  Fulvestrant  Letrozole | 2.223  1.664  2.355  2.113 | 0.788  0.525  0.918  0.828 | 6.272  5.277  6.043  5.395 | 0.131  0.387  0.075  0.118 | 5.155  4.668  5.389  5.463 | 1.091  0.895  1.211  1.233 | 24.36  24.36  23.98  24.19 | **0.038**  0.068  **0.027**  **0.025** | | 0.830  0.546  0.984  1.241 | 0.256  0.132  0.337  0.430 | 2.692  2.259  2.868  3.581 | 0.756  0.403  0.976  0.690 |
| Line of therapy (Ref: ≥ 2L)  1L  2L | 1.059  1.061 | 0.603  0.641 | 1.860  1.756 | 0.842  0.819 | 1.528  1.422 | 0.834  0.818 | 2.801  2.472 | 0.170  0.213 | | 1.348  2.309 | 0.646  1.192 | 2.812  4.472 | 0.427  0.013 |

1L, first-line; 2L, second-line; ECOG PS, Eastern Cooperative Oncology Group performance status scale; EORTC-QLQ-C30, European Organisation for Research and Treatment of Cancer Quality-of-Life Questionnaire Core 30; LCL, lower confidence limit; OR, odds ratio; Ref, reference; UCL, upper confidence limit.

Online Resource 6. Sensitivity analysis of mean scores of the EORTC QLQ-C30 among the 246 patients who completed questionnaires at baseline throughout month 18: a) GHS/QoL b) functional domains c) symptom domains
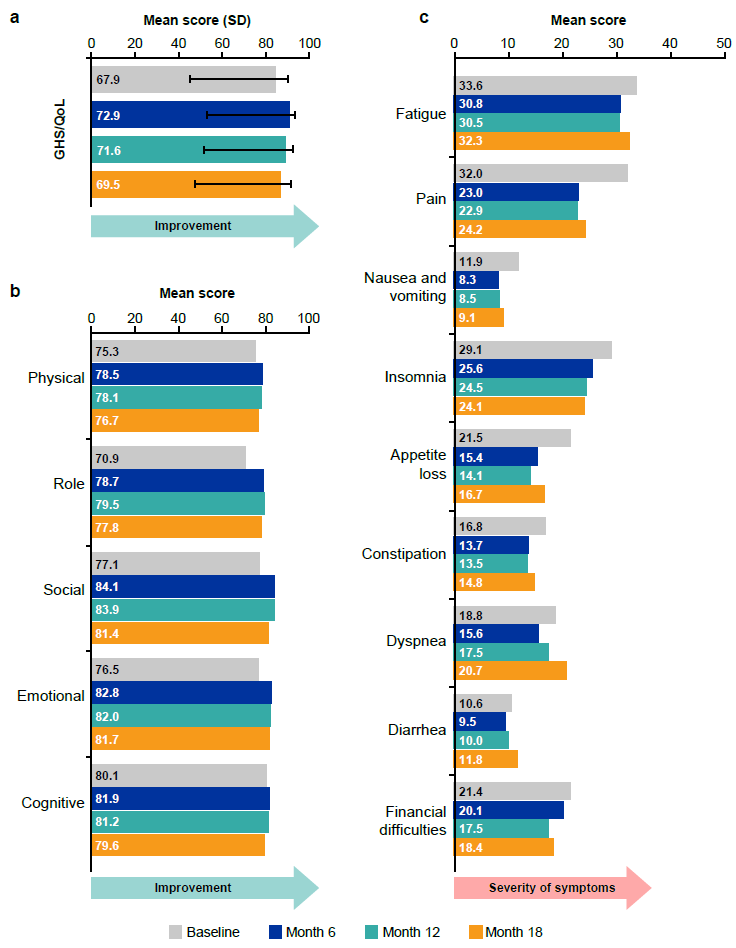


Mean scores were assessed among all patients with EORTC QLQ-C30 data through month 18 (n = 246) at the indicated timepoints. Note: a higher score for global health status/quality of life (GHS/QoL) and functional scales indicates improvement, whereas for symptom scales, a higher score indicates greater severity of symptoms. Error bars represent standard deviation.

**Online Resource 7. Sensitivity analysis among patients who completed EORTC QLQ-C30 questionnaires at baseline throughout month 18: patients with functional scale scores below the clinical importance threshold, suggesting a health problem**

| **Functional scale** |  | **Patients with scores below threshold, n (%)** | | | |
| --- | --- | --- | --- | --- | --- |
|  | **Clinical**  **importance threshold [14]** | **Baseline^a,b^** | **Month 6^a,b^** | **Month 12^a,b^** | **Month 18^a,b^** |
| Physical^c^ | < 83 | 125 (50.8) | 113 (45.9) | 121 (49.2) | 120 (49.0) |
| Role^c^ | < 58 | 72 (29.3) | 44 (17.9) | 41 (16.7) | 43 (17.6) |
| Social^c^ | < 58 | 54 (22.0) | 33 (13.4) | 27 (11.0) | 35 (14.3) |
| Emotional^c^ | < 71 | 89 (36.2) | 58 (23.6) | 62 (25.2) | 64 (26.1) |
| Cognitive^c^ | < 75 | 80 (32.5) | 72 (29.3) | 77 (31.3) | 86 (35.1) |

^a^ Patients who completed EORTC QLQ-C30 questionnaires at baseline throughout month 18; n = 246.

^b^ Percentages for functional scales were calculated based on “n,” the number of patients with functional score measurements available.

^c^ n (missing): baseline, 246 (0); month 6, 246 (0); month 12, 246 (0); month 18, 245 (1).

**Online Resource 8. Sensitivity analysis among patients who completed EORTC QLQ-C30 questionnaires at baseline throughout month 18: patients with symptom scale scores above the clinical importance threshold, suggesting a health problem**

| **Symptom scale** |  | **Patients with scores above threshold, n (%)** | | | |
| --- | --- | --- | --- | --- | --- |
|  | **Clinical**  **importance threshold [14]** | **Baseline^a,b^** | **Month 6^a,b^** | **Month 12^a,b^** | **Month 18^a,b^** |
| Fatigue^c^ | > 39 | 83 (33.7) | 66 (26.8) | 65 (26.4) | 71 (29.0) |
| Pain^c^ | > 25 | 126 (51.2) | 103 (41.9) | 98 (39.8) | 97 (39.6) |
| Nausea/vomiting^d^ | > 8 | 88 (35.8) | 71 (28.9) | 73 (29.7) | 77 (31.3) |
| Insomnia^c^ | > 50 | 48 (19.5) | 43 (17.5) | 36 (14.6) | 35 (14.3) |
| Appetite loss^c^ | > 50 | 46 (18.7) | 20 (8.1) | 16 (6.5) | 27 (11.0) |
| Constipation^e^ | > 50 | 26 (10.6) | 16 (6.5) | 20 (8.1) | 17 (7.0) |
| Dyspnea^f^ | > 17 | 106 (43.3) | 96 (39.2) | 102 (41.5) | 128 (52.0) |
| Diarrhea^g^ | > 17 | 62 (25.3) | 61 (24.8) | 60 (24.4) | 69 (28.2) |
| Financial difficulties^c^ | > 17 | 98 (39.8) | 104 (42.3) | 93 (37.8) | 100 (40.8) |

^a^ Patients who completed EORTC QLQ-C30 questionnaires at baseline throughout month 18; n = 246.

^b^ Percentages for functional scales were calculated based on “n,” the number of patients with functional score measurements available.

^c^ n (missing): baseline, 246 (0); month 6, 246 (0); month 12, 246 (0); month 18, 245 (1).

^d^ n (missing): baseline, 246 (0); month 6, 246 (0); month 12, 246 (0); month 18, 246 (0).

^e^ n (missing): baseline, 246 (0); month 6, 246 (0); month 12, 246 (0); month 18, 244 (2).

^f^ n (missing): baseline, 245 (1); month 6, 245 (1); month 12, 246 (0); month 18, 246 (0).

^g^ n (missing): baseline, 245 (1); month 6, 246 (0); month 12, 246 (0); month 18, 245 (1).

Online Resource 9. Per-label population: patient baseline demographic and disease characteristics

| **Characteristics** | **Patients (N = 861)** |
| --- | --- |
| Age at enrollment, years | |
| Median (range) | 64.0 (22–97) |
| Distribution, n (%) | |
| < 50 | 112 (13.0) |
| 50–69 | 455 (52.9) |
| ≥ 70 | 293 (34.1) |
| Sex, n (%) | |
| Male | 12 (1.4) |
| Female | 849 (98.6) |
| Race, n (%) | |
| American Indian or Alaska Native | 6 (0.7) |
| Asian | 11 (1.3) |
| Black | 99 (11.5) |
| Native Hawaiian or other Pacific Islander | 3 (0.3) |
| White | 709 (82.3) |
| Other | 15 (1.7) |
| Not reported/missing | 18 (2.1) |
| Ethnicity, n (%) | |
| Hispanic or Latino | 61 (7.1) |
| Not Hispanic or Latino | 775 (90.0) |
| Not reported/missing | 25 (2.9) |
| Disease stage at enrollment, n (%) | |
| Locally advanced | 37 (4.3) |
| Metastatic | 822 (95.5) |
| Not reported | 2 (0.2) |
| Sites of distant metastases at ABC diagnosis^a^ |  |
| Visceral disease | 331 (40.3) |
| Bone-only | 299 (36.4) |
| Bone plus other metastases | 331 (40.3) |
| Disposition at enrollment, n (%) | |
| Recurrent from earlier stage, stages 0–III | 589 (68.4) |
| *De novo*, Stage IV at/near initial diagnosis | 237 (27.5) |
| Not reported | 35 (4.1) |
| Time from ABC diagnosis to enrollment, months | |
| Median (range) | 1.1 (0–193) |
| Missing, n | 4 |
| Distribution, n (%) | |
| ≤ 1 month | 406 (47.4) |
| > 1–2 months | 195 (22.8) |
| > 2–6 months | 79 (9.2) |
| > 6 months | 177 (20.7) |
| Line of therapy^b^, n (%) | |
| 1L | 712 (82.7) |
| 2L | 74 (8.6) |
| > 2L | 75 (8.7) |

^a^ Among patients with metastatic disease at enrollment. Visceral disease refers to metastases of the brain, liver, and/or lung/pleura.

^b^ Line of therapy (LOT) is defined as the number of systemic therapies taken after initial diagnoses of advanced or metastatic breast cancer, but before palbociclib treatment start. First-line patients had no LOT before palbociclib initiation.

1L, first-line; 2L, second-line; > 2L, greater than second-line; ABC, advanced or metastatic breast cancer.

Online Resource 10. Per-label population: demographic and disease characteristics of patients who completed the EORTC QLQ-C30 at study months 6, 12, and 18

|  | **Month 6** | **Month 12** | **Month 18** |
| --- | --- | --- | --- |
| **Characteristics** | **(n = 530)** | **(n = 354)** | **(n = 258)** |
| Age at enrollment, years | |  |  |
| Median (range) | 65.0 (29.0–96.0) | 66.0 (29.0–88.0) | 66.0 (29.0–88.0) |
| Distribution, n (%) | |  |  |
| < 50 | 64 (12.1) | 38 (10.7) | 28 (10.9) |
| 50–69 | 278 (52.5) | 192 (54.2) | 134 (51.9) |
| ≥ 70 | 188 (35.5) | 124 (35.0) | 96 (37.2) |
| Sex, n (%) | |  |  |
| Male | 9 (1.7) | 5 (1.4) | 6 (2.3) |
| Female | 521 (98.3) | 349 (98.6) | 252 (97.7) |
| Race, n (%) | |  |  |
| American Indian or Alaska Native | 2 (0.4) | 1 (0.3) | 1 (0.4) |
| Asian | 7 (1.3) | 2 (0.6) | 3 (1.2) |
| Black | 58 (10.9) | 36 (10.2) | 24 (9.3) |
| Native Hawaiian or other Pacific Islander | 1 (0.2) | 1 (0.3) | 0 |
| White | 444 (83.8) | 301 (85.0) | 219 (84.9) |
| Other | 6 (1.1) | 5 (1.4) | 5 (1.9) |
| Not reported/missing | 12 (2.3) | 8 (2.3) | 6 (2.3) |
| Ethnicity, n (%) | |  |  |
| Hispanic or Latino | 31 (5.8) | 21 (5.9) | 15 (5.8) |
| Not Hispanic or Latino | 485 (91.5) | 324 (91.5) | 235 (91.1) |
| Not reported/missing | 14 (2.6) | 9 (2.5) | 8 (3.1) |
| Disease stage at enrollment, n (%) | |  |  |
| Locally advanced | 21 (4.0) | 13 (3.7) | 8 (3.1) |
| Metastatic | 508 (95.8) | 340 (96.0) | 250 (96.9) |
| Not reported | 1 (0.2) | 1 (0.3) | 0 |
| Sites of distant metastases at ABC diagnosis^a^ | | | |
| Visceral disease | 185 (36.4) | 125 (36.8) | 87 (34.8) |
| Bone-only | 191 (37.6) | 128 (37.6) | 93 (37.2) |
| Bone plus other metastases | 202 (39.8) | 127 (37.4) | 94 (37.6) |
| Disposition at enrollment, n (%) | |  |  |
| Recurrent from earlier stage, stages 0–III | 345 (65.1) | 221 (62.4) | 158 (61.2) |
| *De novo*, Stage IV at/near initial diagnosis | 161 (30.4) | 114 (32.2) | 88 (34.1) |
| Not reported | 24 (4.5) | 19 (5.4) | 12 (4.7) |
| Time from ABC diagnosis to enrollment, months | |  |  |
| Median (range) | 1.0 (0–191) | 1.1 (0–191) | 1.0 (0–191) |
| Distribution, n (%) | |  |  |
| ≤ 1 month | 257 (48.7) | 168 (47.6) | 134 (52.1) |
| > 1–2 months | 124 (23.5) | 91 (25.8) | 66 (25.7) |
| > 2–6 months | 50 (9.5) | 38 (10.8) | 23 (8.9) |
| > 6 months | 97 (18.4) | 56 (15.9) | 34 (13.2) |
| Line of therapy^b^, n (%) | |  |  |
| 1L | 446 (84.2) | 304 (85.9) | 227 (88.0) |
| 2L | 44 (8.3) | 27 (7.6) | 18 (7.0) |
| > 2L | 40 (7.5) | 23 (6.5) | 13 (5.0) |

^a^ Among patients with metastatic disease at enrollment. Visceral disease refers to metastases of the brain, liver, and/or lung/pleura.

^b^ Line of therapy (LOT) is defined as the number of systemic therapies taken after initial diagnoses of advanced or metastatic breast cancer, but before palbociclib treatment start. First-line patients had no LOT before palbociclib initiation.

1L, first-line; 2L, second-line; > 2L, greater than second-line; ABC, advanced or metastatic breast cancer.

**Online Resource 11. Per-label population: differences in mean scores for GHS/QoL among questionnaire completers vs non-completers**

| **GHS/QoL completion** | **Difference in means^a^**  **(95% CI)^b^** | ***P*-value^a^** |
| --- | --- | --- |
| Only at baseline vs at baseline and month 6 | −0.5 (−5.2, 4.3) | 0.845 |
| Only at baseline vs at baseline, month 6 and month 12 | −4.6 (−9.8, 0.5) | 0.079 |
| Only at baseline vs at baseline, month 6, 12, and 18 | −7.4 (−11.9, −2.8) | 0.002 |
| At baseline and month 6 vs at baseline, month 6 and month 12 | −4.2 (−9.5, 1.2) | 0.124 |
| At baseline and month 6 vs at baseline, month 6, 12, and 18 | −6.9 (−11.7, −2.0) | 0.006 |
| At baseline, month 6 and 12 vs at baseline, month 6, 12, and 18 | −2.7 (−7.8, 2.4) | 0.294 |

^a^ The difference in means is mean score of fewer measurements (e.g., only at baseline minus mean score of more completed measurements (e.g., at baseline and month 6).

^b^ According to two-group independent sample t-test. CI, confidence interval; GHS/QoL, global health status/quality of life.

**Online Resource 12. Per-label analysis set: mean EORTC QLQ-C30 scores compared with US normative data**

|  | **POLARIS**  Mean score (SD) | **US normative data (n=1009)^e^**  Mean score (SD) |
| --- | --- | --- |
| GHS/QoL | | |
| Baseline^a^  Month 6^b^  Month 12^c^ | 63.8 (23.9)  69.7 (20.8)  71.0 (20.3) | 63.9 (22.9) |
| Month 18^d^ | 69.9 (21.8) |  |
| Functional scales | | |
| Physical  Baseline  Month 6  Month 12 | 72.9 (24.8)  76.8 (22.0)  77.4 (21.6) | 80.8 (25.2) |
| Month 18 | 76.2 (23.5) |  |
| Role  Baseline  Month 6  Month 12 | 71.2 (32.5)  76.3 (27.7)  79.2 (26.0) | 81.7 (28.2) |
| Month 18 | 77.2 (28.0) |  |
| Social  Baseline  Month 6  Month 12  Month 18 | 75.3 (29.3)  81.0 (24.9)  81.8 (25.0)  82.2 (23.1) | 81.6 (29.4) |
| Emotional  Baseline  Month 6  Month 12  Month 18 | 73.8 (23.5)  80.0 (20.3)  80.9 (21.3)  82.0 (17.9) | 73.3 (28.0) |
| Cognitive  Baseline  Month 6  Month 12  Month 18 | 78.6 (24.6)  80.6 (21.3)  81.4 (22.5)  78.5 (22.5) | 80.9 (25.6) |
| Symptom scales | | |
| Fatigue  Baseline  Month 6  Month 12  Month 18 | 36.4 (26.8)  34.5 (22.8)  32.5 (23.7)  32.3 (24.2) | 31.9 (27.8) |
| Pain  Baseline  Month 6  Month 12  Month 18 | 34.8 (31.3)  26.7 (26.2)  25.7 (26.5)  25.1 (27.2) | 27.5 (30.2) |
| Nausea/vomiting  Baseline  Month 6  Month 12  Month 18 | 12.0 (20.9)  9.5 (18.0)  9.1 (16.6)  8.7 (16.7) | 10.9 (22.6) |
| Insomnia  Baseline  Month 6  Month 12  Month 18 | 31.9 (30.7)  28.9 (28.9)  25.8 (28.6)  24.5 (27.1) | 30.8 (33.2) |
| Appetite loss  Baseline  Month 6  Month 12  Month 18 | 22.6 (29.6)  16.5 (24.6)  14.6 (24.1)  16.6 (25.1) | 14.1 (25.3) |
| Constipation  Baseline  6 months  12 months  18 months | 19.2 (27.7)  15.5 (23.2)  14.4 (24.1)  15.1 (21.6) | 18.6 (28.6) |
| Dyspnea  Baseline  6 months  12 months  18 months | 22.9 (29.0)  18.2 (23.7)  17.7 (23.7)  20.3 (24.2) | 19.9 (28.5) |
| Diarrhea  Baseline  6 months  12 months  18 months | 13.2 (23.7)  13.0 (22.7)  10.1 (20.0)  12.3 (22.0) | 13.7 (27.1) |
| Financial difficulties  Baseline  6 months  12 months  18 months | 25.8 (32.7)  22.3 (29.8)  19.3 (27.3)  17.4 (25.8) | 17.5 (30.8) |

^a^ n = 810; ^b^ n = 530; ^c^ n = 354; ^d^ n = 258; ^e^Source of US population norm – Nolte et al, 2019 [17].

EORTC QLQ-C30, European Organisation for Research and Treatment of Cancer Quality-of-Life Questionnaire Core 30; GHS/QoL, global health status/quality of life; SD, standard deviation.

Online Resource 13. Per-label analysis set: mean scores of a) GHS/QoL b) functional scales and c) symptom scales as measured by the EORTC QLQ-C30


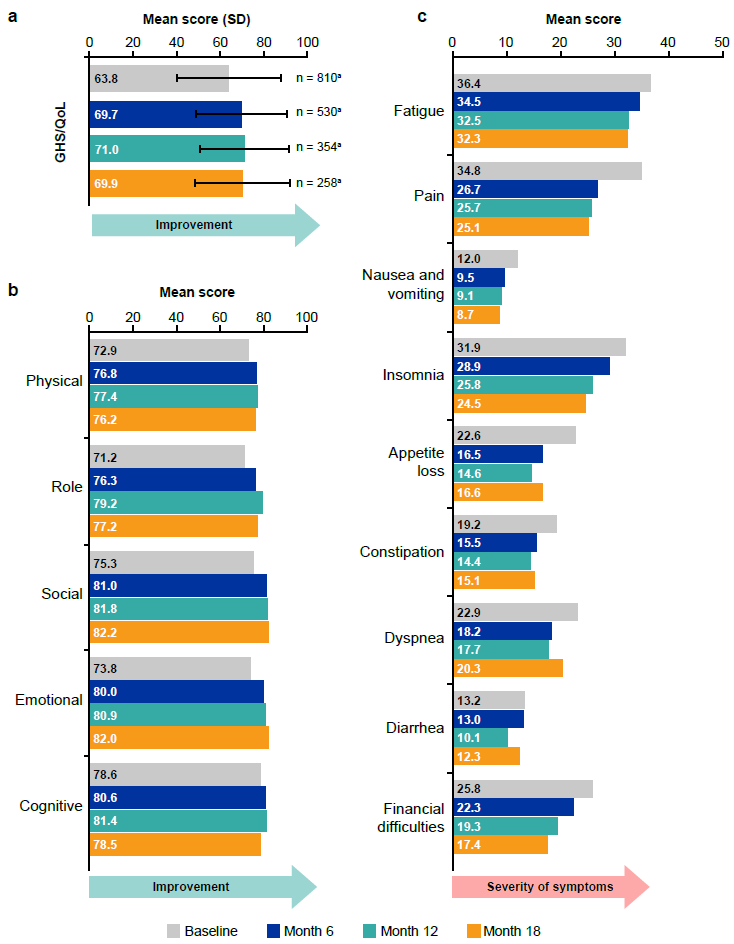


^a^ Mean scores were assessed among the patients with completed EORTC QLQ-C30 questionnaires at the indicated timepoints.

Note: a higher score for GHS/QoL and functional scales indicates improvement, whereas for symptom scales, a higher score indicates greater severity of symptoms. Error bars represent standard deviation (SD). EORTC QLQ-C30, European Organisation for Research and Treatment of Cancer Quality-of-Life Questionnaire Core 30; GHS/QoL, global health status/quality of life.

Online Resource 14. Per-label analysis set: subgroup analyses of mean scores of GHS/QoL


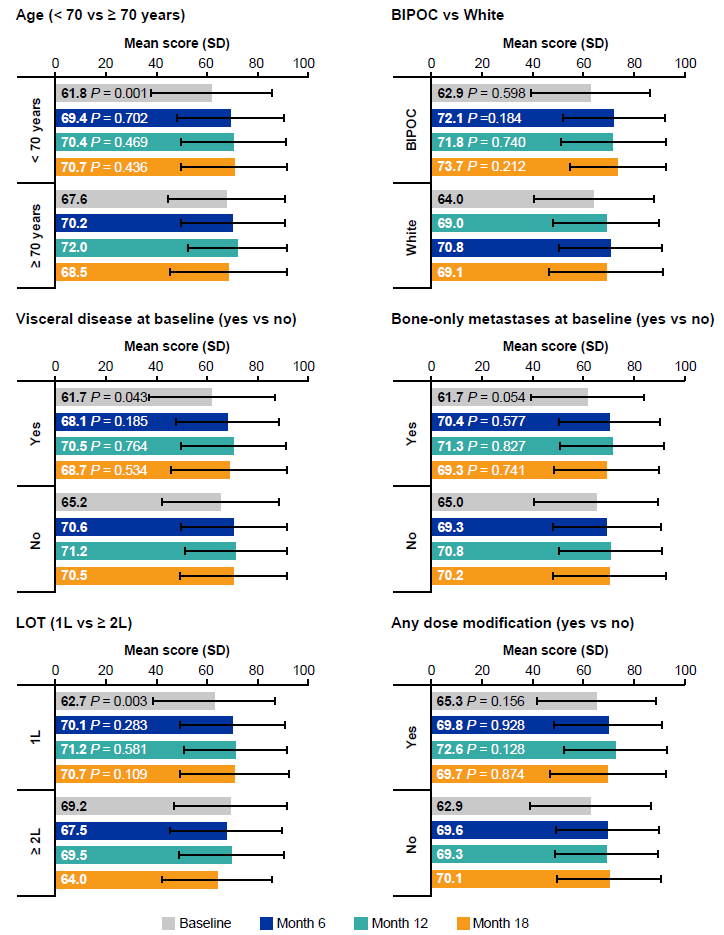


Error bars represent standard deviation. Significant differences in GHS/QoL mean scores of the subgroups at each time point were determined based on two-group independent sample t-tests, with the corresponding reflecting the difference between subgroups at that particular time point. Mean scores were assessed among patients with completed EORTC QLQ-C30 questionnaires at the indicated timepoints.

Age – baseline: < 70 years, n = 524; ≥ 70 years, n = 286; Month 6: < 70 years, n = 342; ≥ 70 years, n = 188; Month 12: < 70 years, n = 230; ≥ 70 years, n = 124; Month 18: < 70 years, n = 162; ≥ 70 years, n = 96

Race – baseline: BIPOC, n = 168; White, n = 618; Month 6: BIPOC, n = 99; White, n = 415; Month 12: BIPOC, n = 62; White, n = 281; Month 18: BIPOC, n = 44; White, n = 206

Visceral disease – baseline: yes, n = 310; no, n = 500; Month 6: yes, n = 185; no, n = 345; Month 12: yes, n = 125; no, n = 229; Month 18: yes, n = 87; no, n = 171.

Bone-only metastases – baseline: yes, n = 287; no, n = 523; Month 6: yes, n = 191; no, n = 339; Month 12: yes, n = 128; no, n = 226; Month 18: yes, n = 93; no, n = 165

LOT – baseline: 1L, n = 669; ≥ 2L, n = 141; Month 6: 1L, n = 446; ≥ 2L, n = 84; Month 12: 1L, n = 304; ≥ 2L, n = 50; Month 18: 1L, n = 227; ≥ 2L, n = 31.

Any dose modification – baseline: yes, n = 321; no, n = 489; Month 6: yes, n = 240; no, n = 290; Month 12: yes, n = 176; no, n = 178; Month 18: yes, n = 139; no, n = 119.

1L, first-line; ≥ 2L, second-line or later; BIPOC, Black, Indigenous and People of Color; GHS/QoL, global health status/quality of life; LOT, line of therapy.

Online Resource 15. Per-label analysis set: mean change from baseline in scores of a) GHS/QoL b) functional domains and c) symptom domains as measured by the EORTC-QLQ-C30


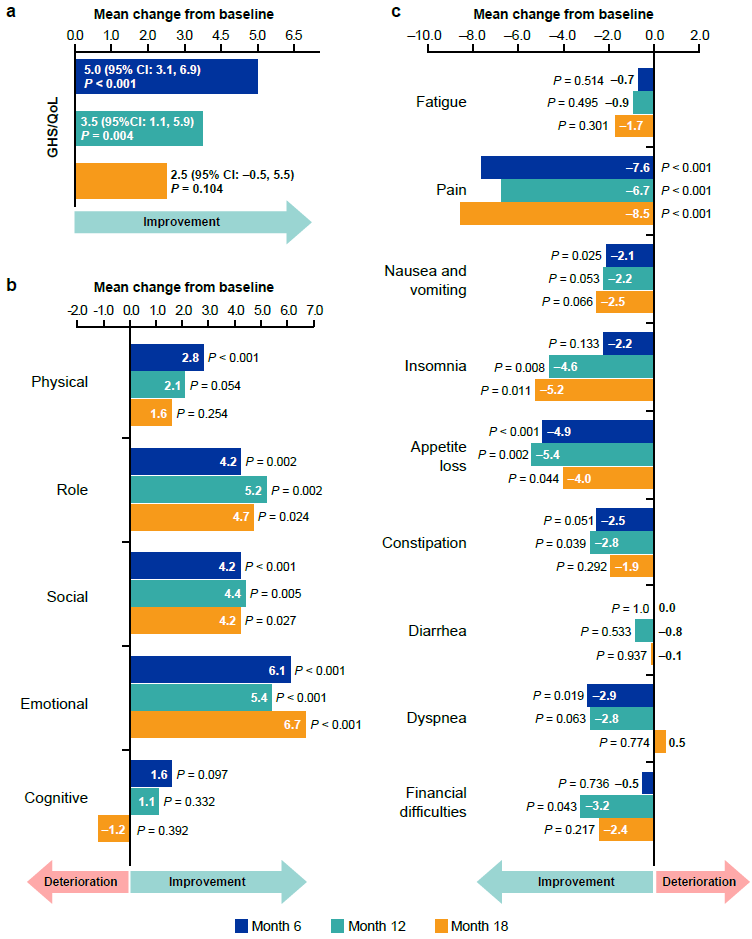


A higher score for GHS/QoL and functional scales indicates improvement, whereas for symptom scales, a higher score indicates greater severity of symptoms (i.e., deterioration). Mean change from baseline represents mean score postbaseline minus mean score at baseline. *P*-values determined from paired t-test of mean changes from baseline. CI, confidence interval; EORTC QLQ-C30, European Organisation for Research and Treatment of Cancer Quality-of-Life Questionnaire Core 30; GHS/QoL, global health status/quality of life.

**Online Resource 16. Per-label analysis set: patients with functional scale scores below the clinical importance threshold, suggesting a health problem**

| **Functional scale** |  | **Patients with scores below threshold, n (%)** | | | |
| --- | --- | --- | --- | --- | --- |
|  | **Clinical importance threshold [14]** | **Baseline^a,b^** | **Month 6^a,b^** | **Month 12^a,b^** | **Month 18^a,b^** |
| Physical^c^ | < 83 | 446 (54.9) | 267 (50.4) | 170 (47.9) | 123 (47.3) |
| Role^c^ | < 58 | 224 (27.6) | 107 (20.2) | 57 (16.1) | 48 (18.5) |
| Social^d^ | < 58 | 185 (22.8) | 78 (14.7) | 52 (14.7) | 34 (13.1) |
| Emotional^e^ | < 71 | 311 (38.3) | 153 (28.9) | 103 (29.1) | 74 (28.6) |
| Cognitive^e^ | < 75 | 280 (34.5) | 180 (34.0) | 106 (29.9) | 98 (37.8) |

^a^ The number of patients eligible at a visit is based on data expected to be available through the latest date of exposure, visit date, or questionnaire date (baseline, n = 861; month 6, n = 770; month 12, n = 695; month 18, n = 600).

^b^ Percentages for functional cales were calculated based on “n,” the number of patients with functional score measurements available.

^c^ n (missing): baseline, 812 (49); month 6, 530 (240); month 12, 355 (340); month 18, 260 (340).

^d^ n (missing): baseline, 811 (50); month 6, 529 (241); month 12, 354 (341); month 18, 259 (341).

^e^ n (missing): baseline, 811 (50); month 6, 530 (240); month 12, 354 (341); month 18, 259 (341).

**Online Resource 17. Per-label analysis set: Patients with symptom scale scores above the clinical importance threshold, suggesting a health problem**

| **Symptom scale** |  | **Patients with scores above threshold, n (%)** | | | |
| --- | --- | --- | --- | --- | --- |
|  | **Clinical importance threshold [14]** | **Baseline^a,b^** | **Month 6^a,b^** | **Month 12^a,b^** | **Month 18^a,b^** |
| Fatigue^c^ | > 39 | 298 (36.7) | 184 (34.7) | 104 (29.3) | 78 (30.1) |
| Pain^d^ | > 25 | 432 (53.2) | 255 (48.1) | 164 (46.2) | 110 (42.3) |
| Nausea/vomiting^d^ | > 8 | 294 (36.2) | 169 (31.9) | 111 (31.3) | 81 (31.2) |
| Insomnia^e^ | > 50 | 195 (24.0) | 108 (20.4) | 63 (17.7) | 40 (15.4) |
| Appetite loss^e^ | > 50 | 139 (17.1) | 50 (9.4) | 35 (9.9) | 29 (11.2) |
| Constipation^c^ | > 50 | 101 (12.4) | 48 (9.1) | 35 (9.9) | 18 (6.9) |
| Dyspnea^f^ | > 17 | 381 (47.1) | 230 (43.5) | 150 (42.3) | 129 (49.6) |
| Diarrhea^g^ | > 17 | 236 (29.1) | 154 (29.1) | 86 (24.3) | 73 (28.4) |
| Financial difficulties^h^ | > 17 | 382 (47.2) | 238 (44.9) | 143 (40.6) | 99 (38.4) |

^a^ The number of patients eligible at a visit is based on data expected to be available through the latest date of exposure, visit date, or questionnaire date (baseline, n = 861; month 6, n = 770; month 12, n = 695; month 18, n = 600).

^b^ Percentages for symptom scales were calculated based on “n,” the number of patients with symptom score measurements available.

^c^ n (missing): baseline, 812 (49); month 6, 530 (240); month 12, 355 (340); month 18, 259 (341).

^d^ n (missing): baseline, 812 (49); month 6, 530 (240); month 12, 355 (340); month 18, 260 (340).

^e^ n (missing): baseline, 811 (50); month 6, 530 (240); month 12, 355 (340); month 18, 259 (341).

^f^ n (missing): baseline, 809 (52); month 6, 529 (241); month 12, 355 (340); month 18, 260 (340).

^g^ n (missing): baseline, 810 (51); month 6, 530 (240); month 12, 354 (341); month 18, 257 (343).

^h^ n (missing): baseline, 810 (51); month 6, 530 (240); month 12, 352 (343); month 18, 258 (342).
